# Molecular actions of sex hormones in the brain and their potential treatment use in anxiety disorders

**DOI:** 10.3389/fpsyt.2022.972158

**Published:** 2022-09-08

**Authors:** Miriam Pillerová, Veronika Borbélyová, Michal Pastorek, Vladimír Riljak, Július Hodosy, Karyn M. Frick, L’ubomíra Tóthová

**Affiliations:** ^1^Faculty of Medicine, Institute of Molecular Biomedicine, Comenius University in Bratislava, Bratislava, Slovakia; ^2^First Faculty of Medicine, Institute of Physiology, Charles University, Prague, Czechia; ^3^Department of Psychology, University of Wisconsin-Milwaukee, Milwaukee, WI, United States

**Keywords:** mood disorders, sex steroid receptors, testosterone, brain structures, molecular mechanism

## Abstract

Anxiety disorders are one of the most prevalent mood disorders that can lead to impaired quality of life. Current treatment of anxiety disorders has various adverse effects, safety concerns, or restricted efficacy; therefore, novel therapeutic targets need to be studied. Sex steroid hormones (SSHs) play a crucial role in the formation of brain structures, including regions of the limbic system and prefrontal cortex during perinatal development. In the brain, SSHs have activational and organizational effects mediated by either intracellular or transmembrane G-protein coupled receptors. During perinatal developmental periods, the physiological concentrations of SSHs lead to the normal development of the brain; however, the early hormonal dysregulation could result in various anxiety diorders later in life. Sex differences in the prevalence of anxiety disorders suggest that SSHs might be implicated in their development. In this review, we discuss preclinical and clinical studies regarding the role of dysregulated SSHs signaling during early brain development that modifies the risk for anxiety disorders in a sex-specific manner in adulthood. Moreover, our aim is to summarize potential molecular mechanisms by which the SSHs may affect anxiety disorders in preclinical research. Finally, the potential effects of SSHs in the treatment of anxiety disorders are discussed.

## Introduction

As specified in the newest (5th) edition of the Diagnostic and Statistical Manual of Mental Disorders (DSM-5), anxiety disorders belong to the category of affective disorders, also known as mood disorders (1), which represent a worldwide health problem (2–4). The global overall prevalence of anxiety disorders is 3.6% with as much as 4.6% among women and 2.6% among men, with differences between age groups 18 and over (3, 5). The most widespread treatment for anxiety disorders and their related disorders, such as panic attacks or phobia-associated disorders, is a combination of psychotherapy—cognitive behavioral therapy, and pharmacotherapy including the use of benzodiazepines (6), serotonin reuptake inhibitors—SSRI, serotonin-norepinephrine reuptake inhibitors—SNRI (4), and tricyclic antidepressants (7). Although the medications are considered relatively safe, there are several negative side effects (8–11). Therefore, the importance of research focusing on studying novel pharmacological approaches is rising, and one of the considered candidates appear to be the sex steroid hormones (SSHs).

Sex steroid hormones, including progesterone, androgens, and estrogens, shape the brain during important prenatal and perinatal periods of development. This is also known as “organizational windows,” when hormones interact with immature neuronal cells. During these periods, SSHs may cause permanent changes in the structure and function of the brain, resulting in sex-specific differences in cognition, such as masculinization of the nervous system, and behavior (12, 13). The main processes participating in the sexual differentiation of the brain include cell birth, cell death, cell migration, and cell differentiation (14). During the postnatal period, another “organizational window” appears—puberty and adolescence—a point at which SSHs may further shape brain development in a way that has long-term effects on behavior in adulthood (15, 16). Masculinization of brain structures starts in early development and ends in early adulthood (17, 18). In rodents, prenatal estrogen exposure leads to defeminization and masculinization of the brain and behavior *via* estrogen receptors (ERs), with a protective effect of estrogen binding protein—α-fetoprotein, against estrogens in females (17). On the other hand, in non-human primates and humans, the masculinization and defeminization of genitalia and behavior are established by testosterone (T) metabolite—dihydrotestosterone (DHT) (18). Early androgen signaling *via* androgen receptor (AR) is important for genital masculinization but does not alter juvenile or adult behavior in non-human male primates. An important period for organizational effects of SSHs on the brain structures during prenatal development appears to be the last part of gestation, which is the period significant for synaptogenesis as well (18). Nevertheless, cellular mechanisms facilitating SSHs actions are particularly dissimilar within and between brain regions (19). SSHs are just one of the factors in these highly complex processes, with many variations at different timespoints along the brain development. The final phenotypic outcome thus can vary greatly (19).

In addition to organizational effects, SSHs have activational effects on brain structures, physiology, and behavior, all depending on fluctuations of SSH concentrations during diurnal, circadian, circatrigintan, or circannual cycles. Activational effects of SSHs are acute and reversible, regulating transient physiological and behavioral responses in adults (12, 13). They generally appear post-puberty and act independently or in combination with organizational effects (15). Therefore, both organizational and activational effects of SSHs on the brain could affect behavioral outcomes later in life, particularly in the development of anxiety.

To exert these effects, SSHs must activate the signaling cascade *via* binding to an appropriate receptor, either intracellular, membrane-associated, or transmembrane. The actions of androgens and estrogens can act *via* classical, slow intracellular AR and ERs, or through non-classical membrane-associated AR/ERs, as well as transmembrane G-protein coupled androgen—zinc transporter protein 9 (ZIP9) and estrogen G-protein-coupled estrogen receptor 1 (GPER1) receptors. Slow, classical processes, which last usually several hours, are mediating the transcription of specific genes in the nucleus. On the other hand, fast, non-classical signaling stimulates various extranuclear downstream cascades regulating different cellular responses, such as DNA synthesis, cell proliferation, migration, or survival (20). The dysregulation of physiological concentrations of SSHs during prenatal and early postnatal development may influence the formation of brain structures and lead to various affective, neurodevelopmental, or neurodegenerative disorders (12, 13, 21, 22). The mean onset of anxiety disorder symptoms starts between late adolescence and early adulthood (23). The frequency and risk of mood, neurological, and psychiatric disorders increase with aging along with a decline of SSHs that occurs in both women and men (24). However, there are significant sex differences in their prevalence. Women are twice as likely to suffer from anxiety disorders when compared to men. The higher prevalence may be related to fluctuations of SSHs during different life periods, such as puberty, pre-menstruum, pregnancy, postpartum, and menopause (25). Moreover, it has long been hypothesized that women are more prone to experience chronic stress and to engage in rumination than males (26). In addition, sex chromosome complement (XX vs. XY) can also be a contributing factor. Expression of sex-determining region Y (*Sry*) gene on the male Y chromosome is responsible for testes differentiation and therefore perinatal T secretion. On the other hand, the absence of the Sry gene in the XX genotype leads to the development of ovaries in females. In the recent review of Mir et al. (27), authors discussed the potential contribution of sex chromosome complement-biased sex differences in rodent models of anxiety-like behavior. Here, the X chromosome appears to affect the development of anxiety-like behavior. For more information, please see the review (27). Described associations suggest that SSHs may be contributing factors in the development and pathophysiology of affective disorders (26, 28, 29). Thus, the attention of this review has been focused on trying to understand the effects of SSHs on the brain and behavior in relation to anxiety in both animals (30–32) and humans (32–37).

## Brain regions involved in anxiety disorders

As mentioned earlier, human brain development is influenced by the organizational and activational effects of SSHs in a sex-dependent manner (13, 16). Brain regions including the amygdala (AMY), hippocampus (HIP), prefrontal cortex (PFC), and hypothalamus (HYP) are implicated in emotional processing, stress response, memory, and cognition. The involvement of each can be sex specific. Dysregulation or damage to these structures may result in affective disorders (38). For this review, the article will concentrate on anxiety disorders.

The PFC is implicated in many cognitive abilities that involve socio-emotional and executive functions (39, 40). The ability to plan complex responses, decision-making, attention and memory, speech and language development (41, 42), mental flexibility, attention, self-control, and self-regulation (43) belong to such executive functions. In addition, a reciprocal connection between the PFC and AMY (basolateral) suggests that PFC may be partially implicated in the regulation of anxiety disorder, and impairment of these neural circuits and functional connectivity during development or in adulthood may contribute to the development of anxiety and/or fear in both male and female rodents and non-human primates (44).

The HIP is predominantly involved in learning and memory, but it also plays a role in spatial navigation, emotions, and the regulation of function in the HYP (45). Structural and functional neuroimaging studies have further revealed the significant involvement of the HIP in the pathophysiology of anxiety (46, 47). The dorsal part of the HIP is mostly involved in spatial memory consolidation, whereas the ventral portion of the HIP, which is connected to AMY is mostly involved in emotional memory (46–48). Bannerman et al. (48) showed that lesions in ventral HIP decrease anxiety in light/dark exploration and hyponeophagia tests and do not affect the spatial memory of male rats in elevated T-maze and water maze (48). Moreover, ventral HIP lesions reduce conditioned freezing, thus, not only AMY but also ventral HIP plays a role in contextual fear conditioning, which is a test, related to anxiety-like behavior in rodents (49).

The AMY plays an important role in processing fearful and threatening stimuli, regulation of emotional responsiveness, facial perception (50), as well as emotional learning and memory for example in reaction to stressful or fear stimuli (51–53). Due to its functions, the AMY is associated with anxiety disorders in animals (54) and humans (51). Finally, the HYP is a part of dominant neuroendocrine systems, such as hypothalamus-pituitary-adrenal (HPA) or hypothalamus-pituitary-gonadal (HPG) axes. Both the HPA and HPG axes are implicated not only in the regulation of reproduction, but also in stress response, and both are related to the regulation of mood and mood disorders including anxiety (55–57).

Meta-analysis of neuroimaging studies also confirmed that the PFC, HIP, and AMY are implicated in anxiety disorders (58). Several studies proposed that the symptoms of anxiety may result in part from disrupted activity in the emotional or cognitive centers in the brain (59), such as the PFC (responsible for a higher cognitive and executive function) and parts of the limbic system, especially the HIP, HYP, and AMY where memory and emotion-processing brain regions are located (60–62). These brain regions express several types of SSHs receptors, such as intracellular/membrane-associated androgen, estrogen, or progesterone receptors (AR, ERs, and PRs, respectively), along with the transmembrane GPER1. Interestingly, expression of the transmembrane receptor for androgen—ZIP9 has also been detected in the brain although primarily found in the prostate (63–65).

Furthermore, the perinatal influence of androgens, estrogens, and progesterone on the PFC, HIP, HYP, and AMY in the context of anxiety disorder development will be discussed. Secondly, the molecular mechanisms and potential treatment of anxiety disorders by SSH for adults will be debated.

## Anxiety disorders

According to the DSM-5, anxiety disorders include several conditions that share common features, such as excessive fear and anxiety, generalized anxiety disorder, panic disorder, separation anxiety disorder, selective mutism, medication-induced anxiety disorder, and different phobia-related disorders (social and agoraphobia) (66, 67). Normal or moderate amounts of anxiety naturally occur in a healthy individual.

It has been previously reported that people can use anxiety for self-motivation (so-called anxiety motivation) (68). In addition, Wirtz et al. (69) have shown that anxiety operates on preparedness behavior, and in turn, it can affect precautionary behavior, both positively and negatively (69). On the other hand, pathological anxiety is characterized by excessive, not adequate anxiety response occurring in situations that would not elicit anxiety in healthy people (70). Pathological anxiety is defined by different features, including excessive worry, physiological arousal, and avoidance behavior (70). Patients suffering from pathological anxiety display affective symptoms, including nervousness, frustration, impatience, and fearfulness (71). Cognitively, it is characterized by hypervigilance for threat, poor concentration, and impaired memory (70, 71). Behaviorally, patients with pathological anxiety display higher readiness to respond to danger, restlessness, and agitation (71).

### Preclinical research

Laboratory rodents, such as mice and rats, are by far the most widely used animals in preclinical anxiety research. Behavioral tests such as open field, novelty suppressed feeding, elevated plus maze, light/dark box, and stress-induced hyperthermia, are widely used to identify anxiety-like behavior (72) and to study the effect of anti-anxiety medication/mechanisms.

Prenatal effects of androgen exposure may mirror the organizational effect of T on the development of brain structures (12, 13). During the prenatal development of rodents, androgen excess (hyperandrogenism), present in mothers with polycystic ovary syndrome (PCOS), may result in a higher risk of anxiety-like behaviors in offspring (22). Female offspring of dams with PCOS showed significantly higher levels of anxiety-like behavior using open field and elevated plus maze in comparison to male offspring (21, 73, 74). In another study in which PCOS was induced by administration of DHT prenatally in dams, female offspring in adulthood showed higher anxiety-like behavior in the elevated plus maze and open-field test when compared to male counterparts. The female offspring had up-regulated genes related to anxiety-like behavior, such as adrenoceptor-α-1B (noradrenergic excitatory input) and corticotropin-releasing hormone (*Crh*) receptor 2 (mediator of stress-related behavior) in the AMY (73). Moreover, adult female offspring of dams with a PCOS phenotype had upregulated *Crh* and downregulated its *Crh* receptor 1 in the HYP. In adult male offspring, the expression of *Crh* was higher only in a group with a high-fat, high-sucrose diet for dams, but not in the PCOS group, thus suggesting sex-dependent mechanisms behind the onset of anxiety-like behavior based on high-androgen excess (73).

The higher prenatal concentration of androgens may also affect the expression of genes, such as brain-derived neurotrophic factor (BDNF) in the brain, especially in females. BDNF and its high-affinity receptor—tropomyosin receptor kinase B (TrkB) in the brain are responsible for the development, differentiation, and survival of neurons, growth of neurites, synaptic plasticity, synthesis of differentiating factors, and homeostasis. They are also involved in responses to social stress and modulate affective-related behaviors (75–78). The western blot analysis showed increased BDNF in HIP and cortex of anxious offspring exposed to higher prenatal androgen concentrations compared to control female offspring of vehicle-treated Wistar rats (22). The increased BDNF concentrations may be an adaptive response of the brain to inhibitory neuron reduction caused by higher prenatal androgen concentrations (22). Experimental studies suggest a link between impaired hippocampal BDNF systems and anxiety disorders. High prenatal androgen excess may lead to dysregulation of neuropeptide Y, parvalbumin, and BDNF, leading to increased anxiety-like behavior. A potential mechanism for this increase may be elevated BDNF concentrations in the HIP and cortex and decreased neuropeptide Y and parvalbumin interneurons in the CA1 region of the HIP (22). Higher concentrations of BDNF in the HIP may be due to adaptive reactions of the brain to the prenatal androgen-induced reduction of hippocampal inhibitory neurons in female rats. It has been shown that inhibition of T aromatization in the brain during the prenatal period *via* the administration of aromatase inhibitor leads to a decrease in basal activity of the hypophyseal-adrenocortical system in male offspring (PND 10) and longer hormonal stress responses in both sexes of adult rats (79). Regarding the behavior of rats, prenatal hyperandrogenization resulted in higher anxiety in the open field and elevated plus maze test in both sexes of adult rats (79–81). Consequently, maternal hyperandrogenization alters the sex-dependent anxiety and the behavioral response to a novel environment in both male and female adult rats (81). In addition, Cheng et al. (82) have shown that a hyperandrogenic intrauterine environment induced by aromatase inhibitor—letrozole—resulted in higher anxiety-like behavior in adolescent rats of both sexes, and it was associated with decreased neurogenesis in the hippocampal dentate gyrus (82).

On the other hand, perinatal (during lactation) exposure to endogenous disruptors (having an anti-androgenic effect) led to higher anxiety-like behavior in elevated plus maze test in young males (postnatal day, PND 45 and 60); however, no changes in anxiety-like behavior of perinatal endogenous disruptor-exposed females were detected at the same age. In addition, in males perinatally exposed to endogenous disruptors, a lower serum concentration of T was found (PND 60). It has been suggested that the anti-androgenic action of endogenous disruptors could be one of the possible mechanisms underlying anxiogenic-like behavior produced by perinatal endogenous disruptor exposure in 60-day-old male rats (83). In contrast, Xu et al. (84) have shown that perinatal (GD 7-PND 21) exposure to endogenous disruptors results in higher anxiety-like behavior in the elevated plus maze and light/dark box in both pubertal males and females, suggesting that exposure to endogenous disruptors in the perinatal period and not just during lactation (as reported by Carbone et al. (83)) has anxiogenic effect in pubertal offspring regardless of sex (84). Furthermore, while in pubertal males, perinatal exposure to endogenous disruptors reduces expression of hippocampal AR, in pubertal females, lower expression of hippocampal ERβ was observed.

It has been shown that an insult, such as prenatal stress, during gestation can reduce the concentration of fetal testicular T in male offspring (85) and, in turn, it can induce a long-term imbalance in circulating T concentrations in males in adulthood (86). It has been demonstrated that prenatal stress leads to a reduction of ERα expression in PFC and HIP of adult male offspring (86, 87). Behaviorally, prenatal stress results in a robust anxiogenic response in adult male rats (88–90) or both sexes of rats (91, 92) and mice (93) in elevated plus maze test in comparison to prenatally non-stressed animals that might be related to reduction in GABA A (benzodiazepine) receptor levels in HIP and AMY observed in prenatally stressed animals (91). Assuming that stress induced during the gestation period leads to higher anxiety-like behavior in male adult offspring (88–90), prenatal impairment of T metabolism might also be related to the anxiogenic responses of male offspring in adulthood.

In conclusion, there is a sex-dependent response to high prenatal effects of androgens, with a higher risk of anxiety-like behavior in female offspring in adulthood caused by upregulation of genes associated with stress response in the AMY and HIP. Although estrogens and progesterone may also be involved, there is not yet enough literature on the effects of prenatal dysregulation of these hormones and/or their metabolites on the brain, and on cognitive or behavioral consequences later in life.

### Clinical research

In humans, it is difficult to directly measure the T concentrations during prenatal development. The second-to-fourth digit ratio (2D:4D) is defined as the ratio of the lengths of the second digit (the “index” finger, 2D) and the fourth digit (the “ring” finger, 4D) of the same hand (94). In men, the 2D is shorter than 4D, while in women, 2D is the same length or longer than 4D. Thus, in females, in general, the 2D:4D is higher than in males (95). The 2D:4D is frequently used as an indirect and non-invasive marker of prenatal T exposure (95), as it has been shown that a lower 2D:4D is the result of higher intrauterine exposure to T, while a higher 2D:4D ratio reflects a lower exposure to T (i.e., higher estrogen exposure) *in utero* (96). Additionally, the fetal concentrations of luteinizing hormone, 17β-estradiol (E2), and prolactin are positively associated with the 2D:4D ratio (97, 98).

Several studies reported that males with feminine finger ratios score higher on a test for anxiety (99) in adulthood. The mechanism behind remains unknown, but it is suggested that high prenatal T with low 2D:4D positively correlates with CAG repeats (coding amino acid glutamine) for AR (100). In turn, the higher number of CAG repeats in AR is related to a less pronounced effect of T response and lower gene transcription. Subsequently, the anxiety level is positively associated with the length of CAG repeats in AR in men (101). Indeed, there are some contradictory results (102); however, at least a portion of the variation in psychopathology is believed to be due to the organizational effects of sex hormones. Thus, the 2D:4D might help to assess the relationship between prenatal androgen factors and a variety of behaviors, including anxiety.

Similar to animal studies, the effect of high prenatal T concentration on the increased risk for anxiety-like behavior in females, but not in male offspring (21, 73, 74) was observed in humans (103, 104). In one recent study, daughters of mothers with PCOS had a 78% risk increase of anxiety disorders compared to daughters of mothers without PCOS, whereas no significant increase in risk was observed in sons (103). In men, a low 2D:4D ratio was significantly associated with higher trait sociability, bigger personal social capital, and a larger personal social network size rather than social anxiety disorders (104). Together, these data support a sex-dependent effect of high prenatal T exposure, which increases the risk for anxiety disorders, particularly in adult women. Clinical studies on the effects of prenatal dysregulation of estrogens and progesterone and/or their metabolites on the brain, and on cognitive or behavioral consequences in adulthood, are lacking.

## Molecular mechanisms of sex steroid hormones action implicated in the regulation of anxiety-like behavior

In multiple animal and human studies, the anxiolytic effects of SSHs were observed through classical signaling pathways of SSH receptors, such as the AR (105, 106), ERs (31, 107–110), and PRs (30, 111). However, non-classical signaling *via* transmembrane receptors, such as GPER1 also appears to play a significant role in the pathophysiology of anxiety disorders in both males and females. The negative correlation of classical effects of E2 was contrasted with the positive correlation of non-classical effects of E2 *via* GPER1 in anxiety disorders (107, 112, 113) in both sexes. The concentration of GPER1 in serum is higher in patients with anxiety than in the controls. Therefore, in humans, GPER1 protein could be a possible candidate for a peripheral biomarker in anxiety disorders (113). Unlike humans, the effect of GPER1 signaling on the anxiety-like behavior of rodents appears to be mostly anxiolytic (112, 114, 115). The relationship between the transmembrane AR ZIP9 and anxiety disorders has not yet been described. Nevertheless, its expression has been detected not only in the gonads but also in the brain (64), suggesting a potential role of the AR ZIP9 receptor in the development of affective disorders. Yet, this is the subject of future research.

### The role of sex steroid hormones receptors in the regulation of anxiety-like behavior

The outcomes of the animal studies lead to the conclusion that high concentrations of prenatal T may lead to a higher risk of anxiety-like behavior mostly in female offspring in adulthood (21, 22, 73). Anxiety-like behavior in female offspring after prenatal androgen exposure as well as intra-AMY microinjections of T appears to be mediated by the effect of T *via* AR on the fetal amygdala (21). These studies also showed a sex- (females) and region-specific (AMY) anxiogenic role of T *via* AR (21, 22, 73).

Activation of ARs appears to have anxiolytic effects in male rats, where the effects of T are blocked with an intramuscular administration of flutamide (selective antagonist of AR). The relationship between the classical effect of T *via* AR and anxiety appears to be non-linear (116). In male rats, the anxiolytic effect *via* AR signaling pathways could be partially mediated by intrahippocampal administration of the T metabolite DHT (117). The anxiolytic effect of the AR signaling pathway has been also supported in the study using AR-knockout male mice (allele altered by recombination using CreLox technology resulted in non-functional AR due to premature termination of AR transcription) since these males show higher anxiety-like behavior than control mice (106). In males, the role of ARs in anxiety appears to be region specific. Although the anxiolytic effect of T *via* AR signaling was observed in the basolateral AMY and suprachiasmatic nucleus of the HYP, no effect was observed in the HIP, medial PFC, paraventricular nucleus of the HYP, bed nucleus of the stria terminalis, or dorsal periaqueductal gray regions (106). Conversely, in male studies comparing wild-type and AR deficient male rats gonadectomized right after birth, gonadectomized rats exhibited lower anxiety-like behavior in both groups compared to their gonadally intact male counterparts. No significant differences between wild-type and AR-deficient male rats indicate that aromatization to E2 rather than AR signaling mediates anxiogenic effects in males (118).

The results from rodent studies on the role of ERα signaling in anxiety-like behavior are inconsistent. In both males and females, ERα signaling has some or no effect on anxiety-like behavior (119, 120). This mechanism may be potentially explained by the action of ERα in the HYP (especially the HPA system), as both intra-paraventricular and peripheral administration of ERα agonist leads to a higher concentration of adrenocorticotropin hormones. These outcomes suggest that ERα may regulate anxiety-like behavior in both male and female rodents *via* stimulating the function of the HPA axis (120).

On the other hand, the studies using ERβ and ERα selective agonists showed that anxiolytic-like behavior is mediated *via* ERβ signaling in adult female mice (8–10 weeks or 7–8 months old) (31, 107) and rats (2–3 months old) (109). ERβ knockout mice exhibited higher anxiety-like behavior in the open field test and elevated plus maze, an effect that was predominant in female mice (107, 121). A reduction of anxiety-like behavior through the ERβ signaling pathway was shown in ovariectomized female mice infused with ERβ selective modulators into the HIP (122) or subcutaneously administered with ERβ selective agonist diarylpropionitrile (109). The signaling *via* ERβ may modulate anxiety-like behavior in female mice *via* its effect on serotonergic or dopaminergic systems. ERβ KO female mice had significantly reduced concentrations of serotonin in numerous regions of the brain (HIP, the bed nucleus of stria terminalis, preoptic area), together with a reduction of dopamine and dihydroxyphenyl acetate concentrations in the striatum (121).

Moreover, it is possible that gamma-aminobutyric acid (GABA) signaling (the transcription/post-translational modifications of GABA A receptor subunit/regulation of the transmembrane chloride gradient) may be influenced by the loss of ERβ in ERβKO female mice with anxiety-like behavior, causing functional changes (53, 107). This would suggest that ERβ may play a crucial role in the manifestation of anxiety-like behavior in female mice by affecting the GABA A receptor (107). In addition, the ERβ signaling pathway may significantly reduce E2 deficiency-induced anxiety-like behavior in female mice through the modulation of “nucleotide oligomerization domain-like receptor family, pyrin domain-containing 3 inflammasome” (110). ERβ signaling has been suggested to be involved in the synthesis of serotonin in males as well as females. The concentration of serotonin precursor (5-hydroxytryptophan) was reduced in the frontal cortex and striatum in ERβKO male mice (123). Regarding the role of ERs on the HPA axis, unlike ERα, ERβ agonist administration into paraventricular nuclei decreases the concentration of adrenocorticotropin hormones in male rats. Furthermore, the peripheral administration of ERβ agonist decreases adrenocorticotropin hormones in ovariectomized mice. These results indicate that ERβ may regulate anxiety-like behavior *via* its inhibitory effects on the function of the HPA axis in both male and female rodents (120).

In humans, higher *Crh* RNA levels were detected in depressed patients postmortem when compared to matched controls. This is accompanied by a higher expression of ERα, involved in the activation of neurons releasing CRH (124). Promoters for the *Crh* gene include estrogen-responsive elements that start the transcription of this gene (125), and androgen-responsive elements, which repress it (126, 127). The results of the above-mentioned studies suggest that disturbed balance in receptor expression could lead to activation of the HPA axis in patients with anxiety.

### The role of non-sex steroid receptors in anxiety disorders

While estrogens regulate anxiety predominantly through ERβ receptors, progesterone (P4) and its metabolites allopregnanolone (ALLO) and pregnanolone appear to promote anxiolytic behavior in female and male rodents through GABA A system (128–132). In the study of Reddy et al. (132), female mice with both a null mutation in the PR gene and wild-type mice had increased concentrations of ALLO in plasma and exhibited lower anxiety-like behavior in the elevated plus maze test. Moreover, pre-treatment of PRKO female mice with the inhibitor of 5α-reductase (finasteride) that blocks the P4 conversion to ALLO, led to higher anxiety-like behavior and, thus, prevented the anxiolytic effect of ALLO (132). P4 and its metabolites can directly modulate GABA A receptor activity *via* the steroid-binding site of the receptor. It has been also shown that GABA A receptor antagonist (picrotoxin) blocks the anxiolytic effect of P4 in ovariectomized rats (129). Another metabolite allosteric modulator of GABA A—pregnanolone showed to have an anxiolytic effect in adult male mice (CFW) (128). Therefore, these data suggest that the anxiolytic effects of P4 may be mediated *via* allosteric modulation of GABA A receptors by P4 metabolites (ALLO or pregnanolone) rather than PRs, although PRs may modulate the anxiolytic response in both males (128) and female rodents (129, 132).

Another system involves BDNF. BDNF is responsible for the development, differentiation, and survival of neurons, growth of neurites, synaptic plasticity, synthesis of differentiating factors, and homeostasis as well as modulation of affective-related behaviors (75–77). There is evidence that higher prenatal concentration of androgens may result in a higher risk of anxiety-like behavior in female but not male offspring later in life (21, 73, 74). The molecular mechanisms behind this process are so far unresolved. One possible mechanism leading to anxiety-like behavior involves the BDNF/TrkB system in both male and female rodents. Specifically, female rats with anxiety-like behavior had increased BDNF concentration in the HIP and cortex and decreased neuropeptide Y as well as parvalbumin interneurons in the CA1 region. This may be caused by adaptive reactions of the brain to the prenatal androgen-induced reduction of hippocampal inhibitory neurons in anxious female rats (16). On the other hand, decreased expression of *Bdnf* in AMY leads to anxiety-like behavior in male rats (111). The effect of higher *Bdnf* expression on anxiety-like behavior appears to be dependent on the brain regions as well as sex (21, 73, 74, 133). Chen et al. (134) reported higher anxiety-like behavior in knock-in *Bdnf^Met/Met^* mice confirming the results of clinical studies (134). Moreover, the administration of fluoxetine, SSRI, failed to normalize higher anxiety-like behavior in these mice supporting that the actions of SSRI require BDNF (135, 136). Moreover, the study of Dincheva et al. (137) reported higher anxiety-like behavior in young adult male *Bdnf^Met/Met^* mice; however, these mice exhibited normal behavior in the adolescent period (137). In addition, the administration of fluoxetine in the early adolescent period led to lower anxiety-like behavior in adult male *Bdnf^Met/Met^* mice, while its administration in the later phase of adolescence or adulthood failed to decrease anxiety-like behavior in adulthood. These results indicate that (a) *Bdnf* Val/Met allele is crucial in the modulation of anxiety-like behavior, (b) the impact of the *Bdnf^Met/Met^* polymorphism or disruption of BDNF signaling occurs in adolescence, and (c) there is a critical time period when fluoxetine administration can normalize higher anxiety-like behavior in adult *Bdnf^Met/Met^* mice (137).

Similarly, as in animals, lately a single nucleotide polymorphism of the *Bdnf* gene (Val66Met) has been identified in humans as a risk factor for anxiety disorders, including post-traumatic stress disorder (134, 138, 139). In addition, the BDNF Val66Met polymorphism appears to be associated with higher trait anxiety (140, 141). On the other hand, patients carrying the 66Met variant displayed stronger amygdala activation in response to emotional stimuli in comparison to neutral stimuli, suggesting that mainly 66Met is associated with higher anxiety (114, 134, 141). The neurotrophic factor nerve growth factor, a growth factor, like BDNF, is necessary for sympathetic and sensory neuron survival and maintenance and is involved in the regulation of stress responses *via* the HPA axis (142). SSHs such as T, E2, and P4 are implicated in the regulation of BDNF or nerve growth factor through which they may provide neuroprotection, neuron survival, and also anxiolytic effects in humans (143) and animals (144–148).

In sum, understanding the molecular mechanisms of SSHs’ effects on the brain is important because it would open the way to the development of novel therapeutic agents for affective disorders. The molecular mechanism of SSHs effect in anxiety disorders has been suggested to be sex- and region specific. The anxiolytic effects appear to involve signaling in the HIP, AMY, PFC, or HPA/HPG axes. These structures are altered *via* ERβ in females and AR signaling in males as well as increased BDNF/TrkB and GABA A receptor activation with serotonergic pathways in both female and male rodents and humans.

## Potential targets for the treatment of anxiety disorders

The cause of anxiety appears to be multifactorial and current psychopharmacological treatments have several limitations as are adverse effects, safety concerns, or restricted efficacy (8–11, 149). Thus, the discovery of novel therapeutic agents would be highly beneficial to patients suffering from affective disorders. As such, understanding how SSHs influence anxiety may open new avenues for drug development to move the field beyond traditional anxiolytic treatments.

Findings about the initial positive effect of E2 on osteoporosis development (150), as well as mood disorders (151) in menopausal females, led to the development of postmenopausal hormone replacement therapies. Unfortunately, an increased risk of endometrial, breast, and ovarian cancer as well as the risk of coronary artery disease was found, and hormone replacement therapy is no longer recommended (152–155). However, the above-mentioned conditions may be mediated through the E2 signaling, and *via* ERα present in the uterus and mammary gland as was concluded by Liu et al. (156) in their comprehensive review (156). Currently, scientists are trying to find selective agonists for ERβ, as these are not associated with adverse side effects as if treated by the ERα signaling pathway. ERβ pathway can represent a potential therapeutic treatment for postmenopausal women suffering from cognitive deficits and generalized anxiety disorders. Several studies showed ERβ agonists to improve memory (147) and alleviate anxiety-like behavior (157) in young or old ovariectomized mice and rats. The most potent ERβ agonists appear to be cycloheptane-hydroxymethane-based agonists (157–159) and also selective estrogen receptor β modulators (SERBAs), which are highly selective (750-fold) and brain penetrant (120). Nevertheless, novel ERβ agonists are being developed. Regarding the chemical structure of estrogens, which contain four cyclic rings (A–D), the most potent and selective appears to be A–C estrogens without B and D steroid rings (159). Such SERBAs have already been shown to improve memory in ovariectomized young mice. In this experiment, the authors demonstrated the biological efficacy of SERBA that was administered *via* three routes: direct dorsal HIP infusion, intraperitoneal infusion, and oral gavage to young ovariectomized mice. For memory testing, object recognition and spatial memory consolidation tasks were used. Interestingly, the SERBAs enhanced object recognition and spatial memory consolidation regardless of the acute or chronic administration (159). Another study with EGX358 substance (another SERBA) led to the consolidation of the memory in young ovariectomized mice, and additionally improved drug-induced vasodilatation (160). Although the latter studies do not directly contribute to anxiety treatment, the ERβ agonists represent a promising treatment with further research necessary. Concerning the role of GPER1 signaling in anxiety, there is an evidence suggesting increased GPER1 signaling in patients with anxiety (113). Thus, the potential use of pharmacological manipulation of GPER1 as the anxiolytic drug may be considered in future research.

Allopregnanolone effects regarding anxiety in women appear to be more inconsistent. Indeed, in line with animal studies (30, 129, 131, 161, 162), lower concentrations of ALLO, which is a potent allosteric modulator of the GABA A receptor complex, were associated with anxiety disorders (163). Oral P4 administration to healthy women (19–39 years old) in the follicular phase was positively associated with the neuronal reactivity of the AMY. The oral administration of P4 led to higher plasma concentrations of ALLO, which mediated inhibitory actions of GABA and potentiated the acute effects of P4, i.e., increased AMY reactivity as shown by functional magnetic resonance imaging. Nevertheless, this study did not show any anxiolytic effect of ALLO previously observed in animals and even suggested that ALLO may paradoxically increase anxiety due to the increased neuronal activity in the AMY (164). In contrast, neurosteroid ALLO has been recently approved for the treatment of postpartum depression by the U.S. Food and Drug Administration (165), and more studies support that treatment with ALLO or combinations of SSHs and antidepressants, could be potentially useful for alleviating both anxieties in women.

The effect of SSHs on the anxiety of men is ambiguous. Indeed, there are many studies indicating the correlation between low T and higher anxiety (166–168) or anxiolysis (threat vigilance, reward processing, general fear reduction, stress resilience) induced by T described in another review (169). Many other modalities and treatments are under investigation in male anxiety. Nevertheless, of these, only a few consider T or other SSH as a treatment. Low-baseline and high-reactive endogenous T concentrations (HPG-axis reactivity) were associated with a decrease in the severity of social anxiety symptoms, such as social avoidance behavior and social fear in adult men 18–50 years old (170, 171). The treatment with P4 (50 and 100 mg) resulted in decreased concentrations of cortisol and increased concentrations of noradrenaline in plasma together with increased blood pressure (systolic blood pressure after 50 mg; diastolic blood pressure after 100 mg). These changes resulted in the attenuation of stress-induced increase in anger and frustration as well as better recovery from negative mood changes after stress exposure probably *via* P4 metabolites (172).

## Discussion

**FIGURE 1:**
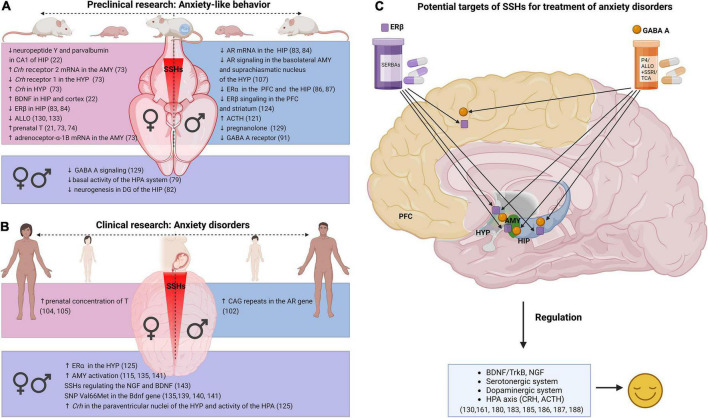
Relationship between SSHs and anxiety in research. **(A)** Dysregulation related to SSHs in anxiety-like behavior—preclinical research, **(B)** dysregulation related to SSHs in anxiety disorders—clinical research, **(C)** treatment approaches for anxiety disorders in adult women and men (Created with BioRender.com). SSHs (sex steroid hormones), AMY (amygdala), Crh (Corticotropin-releasing hormone), HYP (hypothalamus), BDNF (Brain-derived neurotrophic factor), TrkB (tropomyosin receptor kinase B), HIP (hippocampus), HPA (hypothalamic-pituitary-adrenal axis), ERβ (estrogen receptor β), ERα (estrogen receptor α), ALLO (allopregnanolone), GABA A (Gamma-aminobutyric acid A receptor), T (testosterone), AR (androgen receptor), DG (dentate gyrus), PFC (prefrontal cortex), ACTH (adrenocorticotropin hormone), SNP (single nucleotide polymorphism), NGF (nerve growth factor), P4 (progesterone), SERBAs (selective estrogen receptor β agonists), SSRI (selective serotonin reuptake inhibitors), TCA (tricyclic antidepressants).

The findings from rodent studies suggest that the effects of SSHs on anxiety-like behavior are very complex and context-dependent, determined by the type of SSHs (T, E2, and ALLO), brain region (HIP, AMY, HYP, and PFC), sex, and age (132, 161, 173). SSHs in the pathophysiology of anxiety disorders appear to include signaling in the HIP, AMY, PFC, or HPA/HPG axes *via* ERβ (or GPER1 concerning the antidepressant-like effects in female rats) in females and AR signaling in males. BDNF/TrkB signaling, GABA A receptor, and the serotonergic system can be implicated in both females and males. Also, there is strong evidence of the anxiolytic-like effects of reduced ALLO acting in the HIP of female rodents (129, 131, 132, 161). In the HIP of male rats, the anxiolytic-like effect on behavior appears to be provided *via* either the actions of T metabolites DHT (174) or E2 (175). Furthermore, high prenatal T has sex-dependent effects on anxiety-like behavior in rodents. Female offspring after high-prenatal T exposure have an increased risk of anxiety-like behavior in adulthood compared with male offspring. Understanding the molecular mechanisms of SSHs action in the brain affecting anxiety disorders is crucial for developing potent novel drugs (Figure 1A).

Considering the positive effect of T, E2, or ALLO treatment on anxiety-like behavior in animals (32, 174, 176–181), clinical studies have been performed (Figure 1B). Of the animal studies, one of the most promising SSHs with antidepressant effects is combined treatment with ALLO (129, 177, 180, 182–184). A very promising pharmacological treatment that provides an anxiolytic effect (Figure 1C) appears to be cycloheptane-hydroxymethane-based agonists—SERBA, which are 750-fold more selective *via* ERβ receptors thus minimizing the side effects of non-selective agents (159). Lastly, a combination therapy using ALLO with either SSRI or tricyclic antidepressants or a combination of T with imipramine as well as E2 with agomelatine are being successfully tried in clinical studies (185).

To conclude, the studies discussed in this review indicate that potential treatment agents based on SSHs should not be considered as a universal treatment for anxiety disorders on one side, but rather should be personally tailored because their function is context-dependent (sex, age, hormones concentration, route of administration, dose, timing, health condition, genetic predisposition, type of affective disorder). Nevertheless, there are still gaps in the pathophysiology and molecular basis of anxiety that should be clarified by further experiments and studies.

## Author contributions

MPi drafted the manuscript. VB revised it critically for important intellectual content and interpreting the relevant literature. MPa and VR revised the manuscript critically for important intellectual content. JH and KF substantially contributed to the conception and design of the manuscript. L’T drafted the manuscript, substantially contributed to the conception and design of the manuscript. All authors contributed to the article and approved the submitted version.

## Abbreviations

2D: the second digit (index finger)
4D: the fourth digit (ring finger)
ALLO: allopregnanolone
AMY: amygdala
AR: androgen receptor
BDNF/*Bdnf*: Brain-derived neurotrophic factor (protein/gene)
CRH/*Crh*: corticotropin-releasing hormone (protein/gene)
DHT: dihydrotestosterone
DSM-5: diagnostic and statistical manual of mental disorders
E2: 17β-estradiol
ER: estrogen receptor
GABA: gamma-aminobutyric acid
GPER1: G-protein coupled estrogen receptor 1
HIP: hippocampus
HPA: hypothalamus-pituitary-adrenal axis
HPG: hypothalamus-pituitary-gonadal axis
HYP: hypothalamus
P4: progesterone
PCOS: polycystic ovary syndrome
PFC: prefrontal cortex
PND: postnatal day
PR: progesterone receptor
SERBA: selective estrogen receptor β modulators
Sry: sex-determining region Y
SSHs: sex steroid hormones
SSRI/SNRI: selective serotonin/norepinephrine reuptake inhibitor
T: testosterone
TrkB: tropomyosin receptor kinase B
ZIP9: zinc transporter protein 9.
